# The effect of school-entry age on health is understudied in low- and middle-income countries: A scoping review and future directions for research

**DOI:** 10.1016/j.ssmph.2023.101423

**Published:** 2023-05-02

**Authors:** Janny Liao, Henning Schröder, Elliot K. Chin, Muideen Owolabi Bakare, Ramaele Moshoeshoe, Mónica L. Caudillo, Kerim M. Munir, Jan-Walter De Neve

**Affiliations:** a Heidelberg Institute of Global Health, Faculty of Medicine and University Hospital, University of Heidelberg, Heidelberg, Germany; b Harvard College, Harvard University, Cambridge, MA, USA; c Child and Adolescent Unit, Federal Neuro-Psychiatric Hospital, Enugu, New Haven, Enugu State, Nigeria; d Department of Economics, National University of Lesotho, Lesotho; e Department of Sociology, University of Maryland, MD, USA; f Division of Developmental Medicine, Boston Children's Hospital, Boston, MA, USA; g Department of Pediatrics, Harvard Medical School, Boston, MA, USA; h Department of Psychiatry, Harvard Medical School, Boston, MA, USA

**Keywords:** School-entry age, Relative age for grade, Human capital, Social epidemiology, Low- and middle-income countries

## Abstract

**Background:**

Substantive literature has assessed the impact of starting school at younger ages relative to peers on health in high-income countries (HICs), but there is little evidence from low- and middle-income countries (LMICs). Conclusions drawn from HICs may not apply to different education contexts and health threats. This study maps the empirical evidence on the effect of school-entry age on health in LMICs and identifies directions for future research.

**Methods:**

We conducted a scoping review between August and September 2022 by systematically searching the health sciences, education, economics, psychology, and general sciences literature and included quantitative and qualitative studies. The exposure of interest was relative age for grade defined as starting or progressing through school at a younger or older age compared to peers who are in the same grade. We extracted key characteristics of included studies and summarized their findings. We categorized results into broad health domains which emerged *a posteriori* from our analyses of included studies, including neurodevelopment and mental health, sexual and reproductive health, non-communicable diseases, and nutrition.

**Findings:**

We identified 8 studies from middle-income countries published between 2017 and 2022. Among those studies, we identified 3 quasi-experimental studies using data from Brazil, Mexico, and Vietnam, and 5 observational studies primarily from Türkiye. Children starting school earlier had an increased risk of being diagnosed with attention deficit hyperactivity disorder, earlier sexual debut and cohabitation, adolescent pregnancy, adolescent marriage, and engaged more frequently in risky behavior compared to children who started school later. Pregnant women who started school younger also had fewer prenatal care visits and experienced more pregnancy complications. Although most studies identified negative health consequences from starting school earlier, the evidence for nutritional outcomes, such as overweight and stunting, was mixed. No studies were identified from low-income countries.

**Conclusions:**

Little is known about the health consequences of school-entry age in low-resource settings. Additional research is needed to investigate the impact of relative age for grade, whether and how these effects persist into adulthood, and to inform strategies that can offset potential disadvantages stemming from school-entry cut-off dates.

## Highlights

• Little is known about the effect of school-entry age on health in the context of poverty.
• This review synthesizes for the first time empirical evidence from low-resource settings.
• Being young-for-grade was linked with mostly adverse health outcomes and behaviors.
• Mixed results were observed for nutritional outcomes, such as overweight and stunting.
• The effect of school-entry age on health remains unexplored in low-income countries.

## 1 Introduction

Most governments mandate age thresholds to enter public school (UNESCO Institute for Statistics database, 2022). In Southern Africa, for instance, children are eligible to start public school if they turn six years old by June 30th that school year, such as in South Africa (Government of South Africa, 2002) and Lesotho (Government of Lesotho, 2010). These policies set up a threshold rule for enrollment eligibility into grade one of primary school. Children who turn six years old by the cutoff are eligible to start grade one that school year (early starters), whereas children who turn six years old after the cutoff will only be eligible to start grade one the following school year (late starters). Due to the use of school-entry age cutoff dates, the oldest children are on average approximately 20% older than the youngest children in their grade one cohort and the average difference between the youngest and oldest children is about one year (Bedard & Dhuey, 2006). At this early stage, initial maturity differences in class may have large downstream effects on human capital development later in life (Johansen, 2021). Compared to their older peers, the youngest members score 4–12 percentiles lower in grade four, and 2–9 percentiles lower in grade eight (Bedard & Dhuey, 2006). Furthermore, in Canada and the United States, the youngest members of each grade cohort are less likely to attend university. Nonetheless, the impacts of school-entry age on human capital in adulthood remains theoretically ambiguous.

Early school entry may expose younger children sooner to knowledge acquired at school, with potential to help shape their behaviors in making healthier choices. It may also increase their exposure to school-based governmental programs that provide daily meals (Khera R, 2006; von Hippel et al., 2007) or mental health screening (Murphy et al., 2017). Early school may also help offset childcare expenditures for parents and facilitate parental employment (Choi & Jung, 2017; Halim et al., 2023; Ryu, 2019). Conversely, younger children on average are relatively less mature compared to their older school-entry age peers and subjected to normative age-based variation in behaviors that affect learning and adaptation, as mediated level of inattention. In later grades, early school-entry age children and adolescents may feel more pressure from older peers to engage in risky health behaviors including unprotected early sexual intercourse or substance use. In turn, earlier age exposure to such outcomes may disproportionately adversely impact their developmental trajectories. The potential role of school-entry age in human capital development has sparked an important debate across disciplinary and sectoral boundaries (Dhuey & Koebel, 2022). It is therefore imperative to better understand the impact of existing policies on school-entry age in terms of positive or potential disruptive effects on children's health and behavior outcomes.

To date, empirical evidence generally points to a more adverse picture on health and behavior outcomes among children who are younger at school entry than their peers in the same grade. Young-for-grade children would be at higher risk, for example, of being diagnosed with attention deficit hyperactivity disorder (ADHD) (Layton et al., 2018). In the United States, the rates of diagnosis as well as treatment of ADHD are higher among children born just before a September 1st cutoff date for school entry compared to those born after the cutoff (Layton et al., 2018). The impacts of school-entry age appear to persist into adolescence. In Norway, starting school early points to worse mental health and teen pregnancy (Black et al., 2005). Similarly, a Danish register study examining the effect of relative age for grade on women's risky health behavior showed that being young-for-grade is associated with a greater degree of high risk health behaviors, including a higher probability of abortion and alcohol poisoning during adolescence and earlier births (Johansen, 2021). The Danish data for males do not show a similar impact on alcohol poisoning and fatherhood. School-entry age has also been shown to contribute to intergenerational transmission of health inequalities. In the United States, for example, school-entry age affected female educational outcomes as well as quality of mate choice, with small heterogenous effects on fertility and infant health (McCrary & Royer, 2011). School-entry age policies manipulate primarily the education of young women with greater risk of dropping out of school.

Little is known on school-entry age effects among children and youth in a low-and middle-income country (LMIC) context, despite the fact that about 90% of all children worldwide live in such poor resource settings (United Nations, 2011). Moreover, if school-entry age affects schooling outcomes, there may be large health benefits to novel interventions that offset potential disadvantages stemming from school-entry cut-off dates (De Neve et al., 2015; Ozier, 2018). The impacts of school-entry age on health outcomes may differ substantially in the context of high poverty and poor education and health systems. Larger class sizes, for example, may further increase the age gap and observed differences in maturity levels (D. Evans & Yuan, 2018, p. 30). Poorer teacher training and the challenges of teaching to a larger heterogeneous group of students may accentuate the impact of school-entry age variation on health, behavior, and academic performance, with competing influences on total years of schooling and cultural variation on expectations for female vs. male education. Similarly, households may invest less in children who underperform in class, particularly when there are large opportunity costs of sending their children to school (i.e., when there is high demand for the children's labor to work on the family farm or provide caregiving to sick household members) (R. Evans & Becker, 2009). For example, among children of migrant farm workers, internally displaced, and refugee children in southeastern Türkiye (formerly known as Turkey), girls are likely to be kept back from school as companion caregivers for younger siblings, with attrition of attendance in school in later grades (Erol et al., 2020; Şimşek et al., 2017). To our knowledge, no study has systematically reviewed the empirical literature on the impact of school-entry age on health in the context of poverty. To address this knowledge gap, we searched the literature on the impact of school-entry age on health outcomes and behaviors in LMICs.

### 1.1 Distribution of studies on age factors, schooling, and health by country-income group

**Fig. 1:**
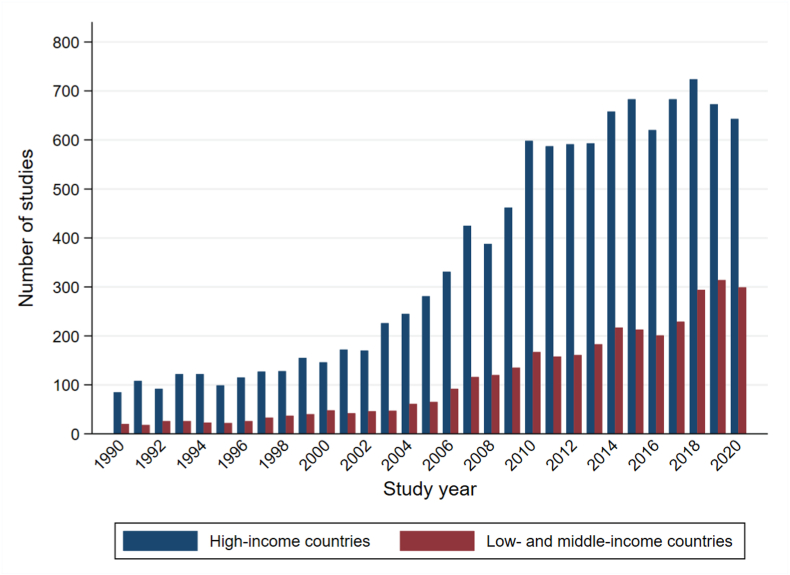
Distribution of articles on age factors and schooling in the health sciences *Notes:* Figure compares the number of articles on “age factors” and education in PubMed over time, separately for high-income countries and low-and middle-income countries. The Medical Subject Headings (or MeSH) term “age factors” was introduced in 1968 and defined by PubMed as “a constituent element or influence contributing to the production of a result. It may be applicable to the cause or the effect of a circumstance. It is used with human or animal concepts but should be differentiated from AGING, a physiological process, and TIME FACTORS which refers only to the passage of time.” Table S1 in the supplementary webappendix provides the complete search history. (Source: https://www.ncbi.nlm.nih.gov/mesh/?term=age+factors).

To illustrate the knowledge gap in evidence between LMICs and high-income countries (HICs), we compared the distribution of documents related to age factors, schooling, and health outcomes between LMICs and HICs over time. To do so, we searched the health sciences for relevant documents using the same search strategy separately for LMICs and HICs. We then plotted the distribution of documents by year and country income group (see Table S1 in the supplementary webappendix for search terms). We used PubMed as an illustrative database because it contains the most comprehensive bibliographic health information and has controlled vocabulary (Medical Subject Headings) for “age factors” and schooling. While all these documents may not strictly be related to school-entry age, this exercise allowed us to compare differences in the distribution of documents related to age, schooling, and health more generally by geographical region. We show these results in Fig. 1. Between 1990 and 2020, the number of documents in the health sciences which assessed age factors and schooling in HICs was, on average, more than 3 times higher compared to the number of documents in LMICs. Specifically, the existing evidence on school-entry age and health adapts a Eurocentric perspective with a strong focus on countries such as Denmark (Johansen, 2021), Germany (Schwandt & Wuppermann, 2016), and Norway (Black et al., 2011). Most studies on school-entry age were done in countries with a relatively structured education system, in which a standardized starting age is set and legally enforced. These HICs often also have detailed data on both demographics and health, allowing longitudinal and quasi-experimental study designs, analyses of mediating pathways, and assessing impacts of school-entry age on health outcomes across the entire life course (Oosterbeek et al., 2021). In contrast, many outcomes and possible mediating or moderating factors which are unique to LMICs appear yet to be explored. The relatively limited evidence on the effects of school-entry age in LMICs has also been observed for education and economic outcomes (as opposed to health outcomes). In a recent evidence map on the impact of school-entry age on education and economic outcomes, fewer than 5% of studies used data from LMICs (Dhuey & Koebel, 2022).

### 1.2 Distinguishing relative age and “pure” age effects

The effects of age can be decomposed into absolute and relative age effects. Absolute age can be defined as age at measurement (“pure” effects or “age-at-test” effects), while relative age can be defined as age with respect to peers in the same grade (Peña, 2017, 2020; Peña & Duckworth, 2018). Children who start school when they are younger, for example, may have worse developmental and mental health outcomes because they are younger (less mature) when they are assessed by their parents or teachers. Conversely, starting school at a younger age compared to peers may also lead to direct and persistent developmental and mental health benefits regardless of absolute age. Empirically, disentangling absolute and relative age effects, however, is challenging because absolute and relative age are usually collinear. To our knowledge, few studies have sought to overcome this collinearity problem by generating independent variation between absolute age and relative age. A Norwegian study, for example, used variation in both school-entry age and outcome measurements at different points in time using a test that was not given simultaneously to all children in the class (Black et al., 2011). Studies have also compared children whose absolute ages are close to each other and therefore relatively similar in terms of background socio-demographic characteristics (including absolute age) but who differ mostly regarding their school-entry age. A quasi-experimental study from the United States, for example, used data on exact date of birth and a school-entry policy to compare the outcomes of individuals who were born within a day of each other but who entered school at different ages (McCrary & Royer, 2011). By focusing on individuals who are born just before and after the school-entry cutoff, this empirical approach further reduces potential differences because of absolute age (such as maturity).

## 2 Methods

We conducted a scoping review, defined as a “preliminary assessment of the potential size and scope of available research literature” (Grant & Booth, 2009). Scoping reviews are distinct from more ‘conventional’ systematic reviews (Munn et al., 2018). While the purpose of a systematic review is to sum up the best available research, the purpose of a scoping review is to map the body of literature on a research question. In this scoping review, our aim was to identify all empirical studies, including published health sciences, education, psychology, demography, economics, and general sciences literature, to map the broad range of the topic, assess heterogeneity in study designs and their key findings, and identify gaps in the existing literature. We considered quantitative and qualitative reports on the effect of school-entry age on health outcomes across the life course in LMICs.

### 2.1 Research question

Our research question was ‘What is the effect of school-entry age on health outcomes among individuals living in LMICs?‘. We adopted an extended definition of the PEO mnemonic (Population, Exposure, and Outcome), including both health outcomes and health-associated outcomes, to design our research question (Munn et al., 2018) consistent with the Joanna Briggs Institute approach to evidence-based healthcare. Based on the research question, we then developed three main concepts for the search strategy, namely: (i) age at school entry; (ii) health outcomes; and (iii) LMICs.

### 2.2 Data sources and search strategy

Guided by the PEO concepts, we developed keywords and controlled vocabulary to search a diverse range of databases, including several field-specific and general sciences databases. First, we conducted a limited search to identify relevant keywords and Medical Subject Headings (or MeSH terms). Second, we searched the electronic databases of PubMed, EconLit, Education Resources Information Center (ERIC), ESBCO, Google Scholar, and the African Education Research Database (AERD). In doing so, we implicitly included the database PsycINFO because it is covered by the EBSCO search database noted above. The search query was in English with no limitations on publication date. We provide the search history of each database in Tables S2–S6 in the supplementary webappendix. Third, we searched for relevant records using the UNESCO Health and Education Resource Centre and relevant conferences proceedings, such as the Population Association of America annual meetings. Fourth, all articles identified as relevant in the search process were included in citation searching. We screened reference lists of included articles to search for additional eligible studies. We also consulted with individuals with relevant expertise and topical knowledge and who may suggest additional references and provide insights beyond those available in the existing literature (Levac et al., 2010; Oliver, 2001).

### 2.3 Study selection

We considered all published empirical studies which report on the effect of school-entry age on health outcomes and behaviors in LMICs as our primary inclusion criterion. The exposure of interest was starting primary school or progressing through school at a younger school enrollment age compared to peers in the same grade (also known as “young-for-grade” and “early starter”) or at an older school enrollment age compared to peers in the same grade (also known as “old-for-grade” or “late starter”). We considered health across the life course and defined health outcomes broadly, including physical health, mental health, and mortality. We also included food security and nutrition, risky health behaviors (such as early unprotected sexual intercourse and substance use) and considered utilization of traditional medicine (such as visiting a traditional healer). To define a country's LMIC eligibility status, we used the World Bank classification. In Box 1, we provide definitions for our study population, exposure, and outcomes. The following criteria led to the exclusion of documents: (i) not related to school-entry age (e.g., because the study focused on school-based health outcomes or using age as a control variable); (ii) not reporting on health outcomes (e.g., because the study exclusively focuses on the effect of school-entry age on educational outcomes such as school performance or years of schooling completed); (iii) study location outside LMICs; (iv) reporting on the opposite causal direction (the effect of children's health on school-entry age as opposed to the effect of school-entry age on health outcomes); (v) systematic review (reference lists of systematic reviews, however, were included in citation searching); (vi) historical article, case study, commentary; (vii) grey literature (e.g., unpublished records such as pre-prints, theses, or working papers in the social sciences); (viii) records that were not published in English; and, lastly, (ix) records that were unrelated to humans.

Box 1 **Population, exposure, and outcome considered in scoping review**

**Table:** 

	Definition	Examples
**Population**	Individuals living in low- and middle-income countries as defined by World Bank classifications.	Brazil India Lesotho
**Exposure**	Starting or progressing through school at a *younger* age compared to peers in the same grade (also known as being “young-for-grade” or an “early starter”)	Starting Grade 1 at 5.5 years old vs. 6.5 years old, both in the same grade, to comply with a local school-entry age cut-off (e.g., turning 6 years old by the middle of Grade 1)
	Starting or progressing through school at an *older* age compared to peers in the same grade (also known as being “old-for-grade” or a “late starter”)	Starting Grade 1 at 6.5 years old vs. 5.5 years old, both in the same grade, to comply with a local school-entry age cut-off (e.g., turning 6 years old by the middle of Grade 1)
**Outcome**	Neurodevelopment and mental health	ADHD, depression
	Sexual and reproductive health	Adolescent pregnancy
	Food security and nutrition	Malnutrition, anemia
	Health behaviors	Smoking, alcohol use
	Healthcare utilization	Primary care visits
	Traditional medicine	Traditional healer visits
	Adult health	Type 2 diabetes
	Mortality	HIV mortality

Alt-text: Box 1

*Notes:* ADHD denotes attention deficit hyperactivity disorder.

### 2.4 Analysis

Our analysis of included studies proceeded in two steps. First, study characteristics were extracted from all included studies, including their author names, year, country, study design, analytical sample, and key findings. We also searched for the journal's Source-Normalized Impact per Paper and percentile ranking within a subject field as a potential indication of study quality and the range of subject fields which published research on school-entry age and health in LMICs. Second, we categorized study findings by broad health domains which emerged from our readings of included studies. These domains included, for example, neurodevelopment and mental health, sexual and reproductive health, and non-communicable diseases. Published studies on other health domains, such as infectious diseases, were not identified.

### 2.5 Conceptual framework

**Fig. 2:**
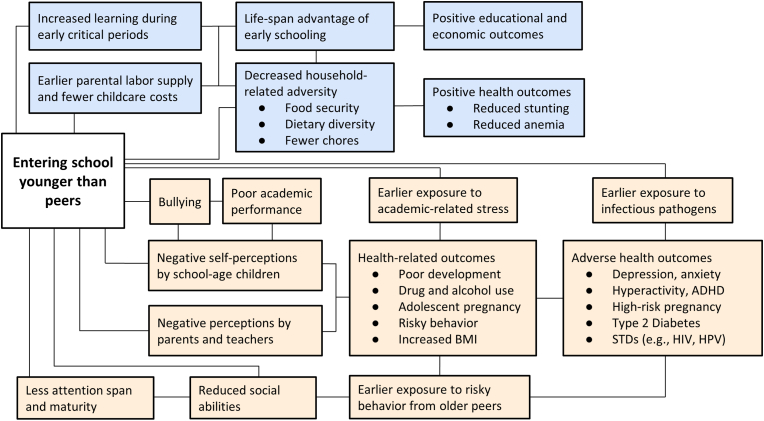
Conceptual framework underpinning the study *Notes:* Conceptual framework for the relationship between school-entry age and health outcomes and behaviors in low- and middle-income countries, categorized into potential positive effects (blue) and negative effects (red). Abbreviations: ADHD: attention deficit hyperactivity disorder; BMI: body mass index; STDs: sexually transmitted diseases. (For interpretation of the references to colour in this figure legend, the reader is referred to the Web version of this article.)

In Fig. 2, we present a conceptual framework underpinning the analysis (Bedard & Dhuey, 2006; Black et al., 2005; Dhuey & Koebel, 2022; Layton et al., 2018; McCrary & Royer, 2011). We hypothesized that the age at which a child enters school may affect human capital development through several pathways. In settings with an upper compulsory schooling age, for example, children who start at a younger age can complete more total years of schooling before reaching the upper compulsory schooling age set by law (e.g., age 15 years). Additional total years of schooling does not only increase an individual's wages (Patrinos & Psacharopoulos, 2020, pp. 53–64; Peet et al., 2015) but also improves a myriad of health outcomes across the life course (De Neve et al., 2015). Similarly, in settings with a compulsory schooling policy based on a specific grade (e.g., having completed at least Grade 9), children who start school younger can complete these compulsory school grades at an earlier age compared to children who start school later. Children who are young-for-grade can then accumulate more total earnings and work experience in the labor market as working-age adults compared to their peers who started school later. Starting school earlier may also reduce total household expenditures on childcare so that more household resources are available to invest in e.g., food and nutritious ingredients. In addition, being young-for-grade may accelerate access to school-based governmental programs which may otherwise not be available to children at home (Murphy et al., 2017). One such example is India's *Midday Meal Scheme*, which provides nutritional support to school children nationwide (Khera R, 2006).

Conversely, starting school early may also have several negative consequences for the development of cognitive skills and learning outcomes in school (Peña, 2020), particularly because school performance is highly correlated with age and level of maturity (Oosterbeek et al., 2021). These negative age effects have been well documented in sports, such as professional soccer, tennis, and ice hockey (Gladwell, 2011). As size, speed and coordination are highly correlated with age, relatively younger players within the same age group or team will on average have poorer performance compared to older peers (Helsen et al., 1998). In turn, the age effects on physical skills can negatively affect the self-evaluation of relatively younger players and the perceptions of their parents and teachers (Thompson et al., 2010). A lower perceived performance by parents and teachers may also lead them to underinvest in students who are relatively younger. These age effects may contribute to changes in body mass index (BMI) (von Hippel et al., 2007) and reduced participation in physical activity in school (Kinyoki et al., 2020). Additionally, being young-for-grade could also lead to more risky health behaviors, such as early sexual debut and substance use, because of peer pressure from older classmates (Johansen, 2021) or higher vulnerability to negative school exposures (e.g., bullying) (Ballatore et al., 2020; Crothers et al., 2010).

## 3 Results

**Fig. 3:**
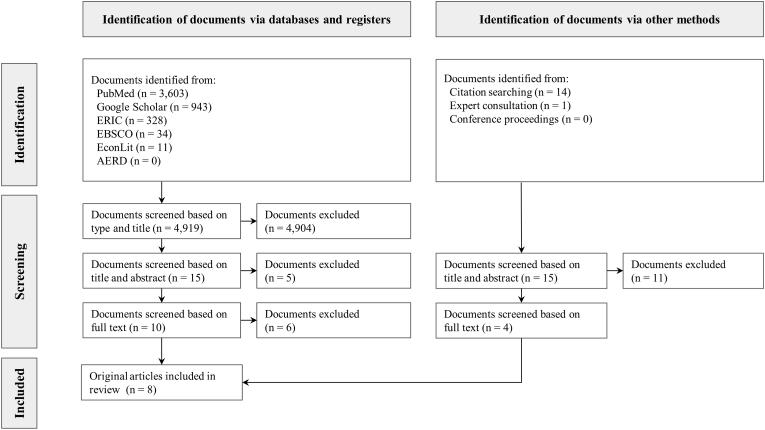
Study selection process Notes: Figure shows a Preferred Reporting Items for Systematic reviews and Meta-Analyses (PRISMA) flow diagram for the identification and screening of studies. Source: Page MJ, McKenzie JE, Bossuyt PM, Boutron I, Hoffmann TC, Mulrow CD et al. The PRISMA 2020 statement: an updated guideline for reporting systematic reviews. *BMJ* 2021; 372:n71.

Our search using databases and registers yielded 4919 documents, catalogued between December 1967 and September 2022, which were screened based on the document type and title. After screening all 4919 documents using the selection criteria described above, we assessed the remaining 15 articles for eligibility based on title and abstract. We excluded 11 studies that did not consider health outcomes (e.g., since they exclusively focused on cognitive development (Dhuey et al., 2019), learning outcomes (Chen, 2017, 2021a, 2021b), or self-esteem (Thompson et al., 2010)), did not use data from LMICs (Berniell & Estrada, 2020; Horstschräer & Muehler, 2012; Mühlenweg et al., 2012), only considered entry into informal education such as kindergarten (as opposed to entry into primary school) (Gottfried & Sublett, 2019; NICHD Early Child Care Research Network, 2007), or were not published at the time of our scoping review (e.g., working paper or thesis (Meng C, 2019)). No systematic reviews or meta-analyses were identified which assessed the effect of relative age for grade on several health domains in LMICs. Most existing systematic reviews focused on the effect of relative age on ADHD symptoms and diagnosis (Caye et al., 2020; Holland & Sayal, 2018; Whitely et al., 2018). We added three studies based on screening the references of studies we identified via databases (Gümüş & Yürümez, 2021; Öner et al., 2019) and one study that was identified through consultation with the research team and experts in the field (Nguyen & Lewis, 2019). Our final sample included a total of 8 articles (4 papers identified via databases and registers and 4 records identified via other methods such as citation searching). We illustrate the inclusion process in a Preferred Reporting Items for Systematic reviews and Meta-Analyses (PRISMA) flow diagram in Fig. 3 (Page et al., 2021).

### 3.1 Study year, population, and design

In Table 1, we show selected study characteristics, including authors (year), country, country income group, data sources, analytical sample, study design, exposure, outcomes, and key findings. All 8 included articles were published within the previous five years. The three of the four articles from Türkiye were published between 2017 and 2019, when the country implemented legislative changes to school-entry age (Gökçe et al., 2017; Öner et al., 2018, 2019). The remaining studies used data from Brazil, Mexico, and Vietnam (Caudillo, 2019; Caye et al., 2020; Levasseur, 2022; Nguyen & Lewis, 2019). All the LMIC studies included therefore were from upper-middle-income economy countries, except Vietnam which is lower-middle-income economy country (Nguyen & Lewis, 2019). There were no low-income economy country studies, and no published studies identified from the African continent. The exposure was generally defined as being young-for-grade (Caudillo, 2019; Caye et al., 2020; Gökçe et al., 2017; Gümüş & Yürümez, 2021; Nguyen & Lewis, 2019; Öner et al., 2018, 2019), with the exception of one quasi-experimental study from Brazil (Levasseur, 2022), which defined the exposure as late enrollment compared to peers in the same grade (as opposed to early enrolment). In terms of outcomes, all 8 studies assessed health outcomes among children and youth aged between 0 and 22 years (Caye et al., 2020). No studies assessed health outcomes among participants older than 22 years. In contrast, studies from HICs have looked at the long-term effects of school-entry age on outcomes throughout adulthood (e.g., ages 30–60 years (Oosterbeek et al., 2021)).

**Table 1:** Studies assessing the effect of school-entry age on health outcomes in low- and middle-income countries.

Authors (year)	Country	Income group	Data source	Analytic sample	Study design	Exposure	Health outcomes	Findings
Caye et al. (2020)	Brazil (urban)	Middle-income	1993 Pelotas Birth Cohort, 2004 Pelotas Birth Cohort, and Brazilian High-Risk Study for Psychiatric Disorders (HRC study)	5249 children up to age 22 years (1993 cohort); 4231 children up to age 11 years (2004 cohort); and 2511 children and adolescents 6–14 years of age (HRC study)	Longitudinal	Young-for-grade	ADHD	Positive association
Caudillo (2019)	Mexico (urban and rural)	Middle-income	2009 and 2014 National Surveys of Demographic Dynamics (ENADID)	3530 women who turned 6 years old between 1997 and 2004	Quasi-experimental	Young-for-grade	Adolescent pregnancy Alcohol Smoking Drug use Pregnancy complications Ever cohabited Ever married	Positive association
Gökçe et al. (2017)	Türkiye (urban)	Middle-income	38 public primary schools	3696 children in Grades 1–2	Cross-sectional	Young-for-grade	ADHD	Positive association
Gümüş and Yürümez (2021)	Türkiye (urban)	Middle-income	Child psychiatry outpatient data	193 children in Grades 1–2	Cross-sectional	Young-for-grade	ADHD	Positive association
Levasseur (2022)	Brazil (urban and rural)	Middle-income	371 public and private schools	16,000 students aged 11–19	Quasi-experimental	Old-for-grade	BMI-for-age Underweight Overweight Height-for-age Stunting	Positive association (stunting) Negative association (overweight)
Nguyen and Lewis (2019)	Vietnam (urban and rural)	Middle-income	2009 Vietnam Population and Housing Census	408,000 girls aged 15–17 and 282,000 girls aged 18–19	Quasi-experimental	Young-for-grade	Adolescent pregnancy and marriage	Positive association
Öner et al. (2018)	Türkiye (urban)	Middle-income	13 primary schools	2058 children in Grade 3	Cross-sectional	Young-for-grade	ADHD symptoms Hyperactivity Impulsivity Inattention	Positive association
Öner et al. (2019)	Türkiye (urban)	Middle-income	10 private primary schools	2627 children in Grades 1–4	Cross-sectional	Young-for-grade	ADHD	Positive association

*Notes:* Table shows selected characteristics of studies assessing the effect of school-entry age on health. The exposure was starting or progressing through school at a younger or older age compared to peers in the same grade. Gökçe et al., 2017 defined the exposure as “early school entry” in Grades 1–2. Türkiye changed its name in 2022 (formerly known as Turkey). All countries are middle-income countries as no eligible studies were identified from low-income countries. See references for citation information. ADHD: attention deficit hyperactivity disorder. BMI: body mass index. ENADID: Encuesta Nacional de la Dinámica Demográfica.

Three out of eight studies used quasi-experimental designs to identify causal effects on health outcomes. A regression discontinuity study, for instance, exploited a government policy in Vietnam that determines the timing of school entry based on date of birth (Nguyen & Lewis, 2019). In Vietnam, school-entry age is 6 years and January 1st is the cut-off for determining a child's school age by law. As a result, children who are born before January 1st enter school one year earlier than children born on or after January 1st. The authors used this government policy to compare the health outcomes of children who were born just before the cut-off (akin to the “treatment group” in a randomized controlled trial) to the health outcomes of children who were born just after the cut-off (akin to the “control group”). Additionally, the authors in the study from Vietnam included age fixed effects in their regression models so that their estimates can be interpreted as the change in health as age decreases by one month within a single-year age group. Our search did not identify qualitative, mixed-methods, or experimental studies.

In Table S7, we provide an overview of all journals which have published studies on school-entry age and health outcomes and behaviors in LMICs and were included in our scoping review. For each journal, we show field-weighted measures of impact and rank within the field. The Source-Normalized Impact per Paper ranged from 0.026 for the *Journal of Experimental and Clinical Medicine (Turkey)* (Gümüş & Yürümez, 2021) to 3.594 for the *Journal of the American Academy of Child & Adolescent Psychiatry* (Caye et al., 2020). Although our scoping review identified few studies, these journals represented a wide range of fields, including medicine, psychology, economics, education, and demography. These findings correspond with studies on school-entry age and health outcomes and behaviors from higher-income settings, which similarly cover a wide range of fields, including, for example, medicine (Layton et al., 2018) and economics (Schwandt & Wuppermann, 2016). It is also worth noting that no studies which were identified in our scoping review were published by journals in several relevant fields, including global health, public health, epidemiology, international health, school health, or journals focused on “diseases of poverty”, such as infectious diseases or neglected tropical diseases (De Neve et al., 2018).

### 3.2 Effect of school-entry age on health in middle-income countries

#### 3.2.1 Neurodevelopment and mental health

Early starters generally reported adverse neurodevelopmental and mental health outcomes compared to late starters (Caye et al., 2020; Gökçe et al., 2017; Gümüş & Yürümez, 2021; Öner et al., 2018, 2019). Children who were young-for-grade were more likely to be diagnosed with ADHD, for example, compared to late starters (Caye et al., 2020; Gökçe et al., 2017; Öner et al., 2019). In a teacher-rated ADHD survey in Türkiye, young-for-grade students were noted to have lower behavior regulation skills and were about three times more likely to be diagnosed with ADHD compared to children who were older-for-grade among primary school students, from first to fourth grades (Gökçe et al., 2017; Öner et al., 2019). The tendency of teachers to rate ADHD symptoms among young-for-grade students is therefore not only limited to HIC settings and the ADHD symptoms loads tend to be higher for younger children. One possible explanation for these observations is that young-for-grade students may experience more neurological vulnerabilities in terms of frontal cortical functioning, such as lesser degree of coordination and fine motor skills, abstraction, attention, behavior regulation and impulse control skills. Such temporal maturational consequences of being young-for-grade may lead to greater likelihood of young-for-grade children being (mis-)classified with neurodevelopmental outcomes. Starting school before being “school ready” may also have genuine physical, mental and social damaging effects among young-for-grade children. An understanding of ontogeny of self-regulation and successful adaptation of children beginning school is likely to mitigate such adverse outcomes (Blair, 2002). Existing studies on neurodevelopment and mental health outcomes in Türkiye and Brazil, however, have generally used observational designs which preclude causal inference in these settings. Nevertheless, the findings from Türkiye and Brazil are generally consistent with estimates from HICs, including from Germany (Schwandt & Wuppermann, 2016) and the United States (Layton et al., 2018) (see Table 2 for a comparison).

**Table 2:** Comparison with selected studies from high-income countries.

*Income group*		Middle-income country		High-income country	
*Country*		Brazil^1^	Türkiye^2^	Germany^3^	United States^4^
*Sample*	*ADHD, % or rate*	1.8–4.5%	9.27%	3.80%	63.6 per 10,000–90.6 per 10,000
	*Description*	Children in three urban areas of Brazil (Pelotas, Porto Alegre, and São Paulo)	Children in urban district with high socio-economic status (Kadıköy, Istanbul)	Publicly insured children (about 90% of German children)	Children with employer-provided insurance (excludes Medicaid and uninsured)
	*Age range (years)*	0–22	5–8	4–14	4–7
	*School grades*	Grades 1–8	Grades 1–2	Grades 1–8	Grades 1–2
*Design*	*Sampling*	Community-based	Community-based	Population-based	Population-based
	*Observations*	11,991	3696	7.2 million	407,846
	*Exposure*	Young-for-grade	Young-for-grade	Young-for-grade	Young-for-grade
	*Study design*	Longitudinal	Cross-sectional	Quasi-experimental	Quasi-experimental
	*Data source*	Parent report	Teacher report	Insurance database	Insurance database
	*Measurement*	ADHD diagnosis	ADHD diagnosis	ADHD diagnosis and medication	ADHD diagnosis and medication
*Findings*	*Results*	Positive association	Positive association	Positive association	Positive association
	*Effect size*	Risk ratio for ADHD comparing youngest vs. oldest children between 1.11 and 2.02	Prevalence of ADHD among early starters (<72 months) was 15.9% vs. 6.4% among late starters (78–83 months)	Risk ratio for ADHD comparing youngest vs. oldest children was 1.24	Rate of ADHD was 85.1 per 10,000 among early starters and 63.6 per 10,000 among late starters

*Notes:* ADHD denotes attention deficit hyperactivity disorder. References: Brazil (Caye et al., 2020)^1^, Türkiye (Gökçe et al., 2017)^2^, Germany (Schwandt & Wuppermann, 2016)^3^, and United States (Layton et al., 2018)^4^.

#### 3.2.2 Sexual and reproductive health

Early school entry has significant implications for early family formation and adolescent pregnancy in MICs. In a quasi-experimental study from Vietnam, being young-for-grade reduced school attendance and accelerated adolescent marriage and motherhood (Nguyen & Lewis, 2019). These effects were qualitatively large. Starting school late reduced teenage motherhood by more than one third among women ages 15–19 years old. Similarly, in a quasi-experimental study from Mexico, being young-for-grade accelerated early sexual debut, pregnancy, and cohabitation among women ages 15–17 years old (Caudillo, 2019). Moreover, pregnant women who were young-for-grade were more likely to engage in drug use, alcohol consumption, and smoking before conception; less likely to attend prenatal care visits; and more likely to have pregnancy-related health complications (such as bleeding excessively during delivery and 40 days postpartum) (Caudillo, 2019). These detrimental effects on maternal health behaviors and outcomes suggest that there may be consequences of mothers’ school-entry age for the next generation, consistent with other studies from HICs (McCrary & Royer, 2011). One possible explanation for these effects of school-entry age on sexual and reproductive health outcomes could be peer effects (Caudillo, 2019; Nguyen & Lewis, 2019). For example, older and more physically mature peers are likely to start engaging in sexual activity, and young-for-grade kids may feel considerable pressure to catch up, even if they are younger in terms of their biological age (Skirbekk et al., 2004). Another possible explanation could be mental fragilities of children who start school earlier compared to peers, including lack of maturity, negative self-perception, and higher vulnerability to negative school exposures such as bullying (Fig. 2) (Ballatore et al., 2020; Crothers et al., 2010). In Vietnam, the deleterious effects of starting school early were particularly pronounced for girls who were members of ethnic minorities, whose mothers had relatively less education, and whose households were relatively poor. These results correspond to findings from HICs, which found that the impact of school-entry age on other adolescent risky behavior (such as criminal behavior (Cook & Kang, 2016)) was generally larger for individuals with relatively less maternal education and from poorer families. These results are important because they suggest that young-for-grade women in these subgroups may benefit most from, for example, delayed school entry, tailored tutoring support, or increased access to sexual and reproductive health programs.

#### 3.2.3 Non-communicable diseases and nutrition

The existing evidence suggests that early school-entry age may negatively affect cardiovascular risk factors. In Mexico, women who were young-for-grade and had ever been pregnant were 9 percentage points more likely to report drinking alcohol, 8 percentage points more likely to report smoking, and 3 percentage points more likely to report having used drugs before their first pregnancy (Caudillo, 2019). In terms of anthropometric measurements, the evidence is more mixed. Instrumental variable estimates from Brazil suggest that being young-for-grade increased the probability of being overweight (defined as a BMI above 25 kg/m^2^) but reduced the probability of being stunted (defined as height-for-age below 2 z-scores) in adolescence (Levasseur, 2022). Specifically, being young-for-grade reduced the probability of being stunted by 1.5 percentage points. One reason for these findings could be that school-entry age policies mediate access to school meals (Gelli et al., 2019; Horta et al., 2019). In Brazil, free meals are offered through the *Programa Nacional de Alimentação Escolar* (Brazilian School Food Program) which started in the 1950s and appears to be one of the largest and most successful school feeding programs worldwide (Locatelli et al., 2018). Meals would be prepared daily in schools, with natural or minimally processed foods, and without products such as sugary drinks and processed meat (Araújo de Sousa et al., 2015). Additionally, increased adolescent overweight in Brazil may be a consequence of risky practices, such as smoking, drinking, and substance use, which are associated with weight gain. Higher vulnerability to negative school exposures such as bullying among early starters (Ballatore et al., 2020; Crothers et al., 2010) may also explain adverse mental health and potential weight gain (Bauman et al., 2013; Black et al., 2011). In Brazil, the effect of delayed school enrollment was particularly detrimental for the nutritional status of students from underprivileged settings (Levasseur, 2022). Late school entry may, for example, increase total childcare expenditures and reduce parental labor supply and earnings so that fewer household resources may be available to invest in food and nutritious ingredients (Fig. 2) (Choi & Jung, 2017). In those cases, earlier school attendance could act as “social protection” for underprivileged families by further alleviating household food pressures (Bakhshinyan et al., 2019).

## 4 Discussion

School-entry age may affect human capital development, but relatively little is known about health impacts in low-resource settings (Dhuey & Koebel, 2022). In this study, we reviewed for the first time, to our knowledge, the published literature on the health effects of school-entry age in LMICs. Despite the large scope of databases which we searched for relevant evidence, we identified just eight relevant studies, published between 2017 and 2022. All studies used quantitative designs and mostly assessed neurodevelopmental and mental health outcomes (Caye et al., 2020; Gökçe et al., 2017; Gümüş & Yürümez, 2021; Öner et al., 2018, 2019). These studies appear largely modelled after earlier studies on neurodevelopmental and mental health outcomes which were conducted in HICs, such as Denmark (Pottegård et al., 2014), Germany (Schwandt & Wuppermann, 2016), Iceland (Zoëga et al., 2012), the United States (Elder, 2010), and the United Kingdom (Goodman & Gledhill, 2003) (Table 2). These existing studies on neurodevelopmental and mental health outcomes focus on drawing parallels with HICs and minimal research has been conducted on outcomes which are unique to LMICs. Only two studies, for example, assessed sexual and reproductive health outcomes (Caudillo, 2019; Nguyen & Lewis, 2019) and only one study assessed nutritional outcomes, such as malnutrition (Levasseur, 2022). The few studies that were available from MICs generally point to a negative association between being young-for-grade and health outcomes in childhood and adolescence, including in Mexico, Vietnam, and Türkiye (Caudillo, 2019; Gökçe et al., 2017; Gümüş & Yürümez, 2021; Nguyen & Lewis, 2019; Öner et al., 2018, 2019). Evidence for anthropometric measurements was mixed in a study from Brazil (Levasseur, 2022).

Across included studies, the most common health outcome assessed was ADHD (Caye et al., 2020; Gökçe et al., 2017; Öner et al., 2019). Lack of attention and hyperactivity was more frequently reported among younger students within the same grade. These relative differences may have been attributed as an ADHD diagnosis, without considering differences in absolute age of children in the same grade or “age at test” (Peña, 2017, 2020; Peña & Duckworth, 2018). These observations may be due to a relative comparison between younger students and their older peers who are emotionally more mature. Additionally, most studies on neurodevelopment and mental health focused on Türkiye (Gökçe et al., 2017; Gümüş & Yürümez, 2021; Öner et al., 2018, 2019). Moreover, two out of four studies from Türkiye were implemented by the same first author and institution (Öner et al., 2018, 2019), potentially limiting the generalizability of these findings to other settings. Early progression through school was also associated with several health risk behaviors and non-communicable disease risk factors, such as tobacco use, alcohol use, and drug use (Caudillo, 2019). Being young-for-grade was also associated with more unprotected early sexual debut (Caudillo, 2019; Nguyen & Lewis, 2019) and adolescent pregnancy, which increased the risk of excessive bleeding during childbirth (Caudillo, 2019).

In Table 2, we show a side-by-side comparison between empirical studies from MICs and selected studies from HICs (Layton et al., 2018; Schwandt & Wuppermann, 2016). We illustrate these comparisons using ADHD as the outcome because it is one of the most studied outcomes in the literature on school-entry age and health (Caye et al., 2020; Holland & Sayal, 2018; Whitely et al., 2018). Studies from higher-income settings also found that being young-for-grade is associated with an increased risk of ADHD (Layton et al., 2018; Schwandt & Wuppermann, 2016). In a German study, for example, the risk ratio was 1.23 (Schwandt & Wuppermann, 2016). Despite several similarities, there were also notable differences between MICs and HICs. In terms of study design, for example, both studies from HICs used quasi-experimental designs. In contrast, studies from MICs used cross-sectional and longitudinal designs, which may make the results from these studies more difficult to interpret (e.g., due to potential residual confounding) and may limit the ability to distinguish relative age from absolute or “pure” age effects (Peña, 2017, 2020; Peña & Duckworth, 2018). Additionally, analyses in HICs were based on nation-wide data from insurance claims (Layton et al., 2018; Schwandt & Wuppermann, 2016). In contrast, studies from MICs used outcomes reported by parents or teachers, which may be vulnerable to recall bias or lead to possible overestimation of the prevalence of ADHD (Gökçe et al., 2017). Studies from MICs also used community-based approaches and focused on urban areas, such as São Paulo (Caye et al., 2020) and Istanbul (Gökçe et al., 2017), suggesting that these results may not apply to more rural areas.

### 4.1 Study limitations

This scoping review brings together studies from a wide range of fields and provides a broad overview of the existing evidence. Nevertheless, it has several limitations. First, our focus on health outcomes and behaviors may underestimate the overall impact of school-entry age, including, but not limited to broader economic, social, and cultural implications (Oosterbeek et al., 2021). These were beyond the scope of the current review. Similarly, although we defined health broadly, including both physical and mental health outcomes, we may have missed outcomes which are closely related to health but were excluded from our review (Peña, 2017). Second, the screening process and data extraction was conducted by one researcher. Nevertheless, in case of doubt, articles were verified by additional study team members until a consensus was reached. Third, as with all literature searches, there is a risk of publication bias. Studies on school-entry age and health may be prone to positive publication bias so that only qualitatively large or statistically significant positive effects may be appealing to publish. No studies on school-entry age and health outcomes included in our review explicitly reported null results as their main finding (Table 1, column 9). Nonetheless, this is not necessarily a bias unique to the current research. Fourth, biological age at start of school is only one way of determining eligibility for primary school. In some contexts, physical measurements as opposed to age are used to determine school eligibility. In Zimbabwe, for example, the “clutch-the-ear” measurement has been a long-established practice (Chilunjika et al., 2020). In this procedure, children use their hand to clutch the ear at the opposite side to the hand (taking the right hand, over the head, to hold the left ear or vice versa). Completion of this task would signify a child's maturity for more demanding tasks such as herding goats and monitoring animal traps. With the beginning of colonization, this approach was used to determine children's readiness for enrollment in primary school. Fifth, our search strategy excluded articles which were not published in English, and we may have missed studies which were published in other languages. Sixth, it was not possible to examine screening procedures in addition to a school-entry age cutoff criterion in each setting. It is likely that screening procedures augmenting school-entry age act differentially by risk group, e.g., excluding those with significant identified medical, cognitive, and adaptive behavioral impairments from early school entry, but including younger for grade children with heterogeneous risk conditions by means of “adverse selection” to lessen parental childcare burden or to benefit from early educational interventions that would otherwise be unavailable to them.

### 4.2 Implications for future research on school-entry age and health in LMICs

Our findings have several implications for future research (Box 2). First, further research is needed to examine the effects of school-entry age in LMICs. The Sustainable Development Goal 4 (SDG 4) mandates inclusive and equitable education and promotion of learning opportunities across the lifespan. Many LMICs have improved educational infrastructure and universal primary education has nearly been achieved. Accordingly, many governments now mandate and enforce school-entry policies (including through fines of parents who refuse to send their children to school) (UNESCO Institute for Statistics database, 2022). Moreover, a wide range of cross-sectional and longitudinal population-based data sources are available which include data on our exposure and outcomes, including Census data (Integrated Public Use Microdata Series, 2020), nationally representative household surveys (Corsi et al., 2012; Madise et al., 2013), and data from Health and Demographic Surveillance Sites (Sankoh & Byass, 2012). These data sources could be used to assess these research questions in other LMIC settings. Second, no published studies assessed the relationship between school-entry age and several health outcomes and behaviors depicted in our conceptual framework (Fig. 2). For example, infectious diseases (e.g., HIV, HPV), most non-communicable diseases and their risk factors (e.g., lack of dietary diversity and anemia), as well as violence and injuries were not assessed. In a recent presentation at the Population Association of America annual meeting, for example, entering school late compared to peers in the same grade substantially reduced HIV infection risk among men in Lesotho, which has the second highest HIV prevalence in the world (De Neve et al., 2023). The current emphasis on specific neurodevelopment and mental health outcomes (such as ADHD) is not distributively aligned with the disease burden in LMICs and mirrors studies from high-income settings (Table 1, Table 2). Although this is a welcome contribution and the results are generally consistent with HIC findings, other outcomes and pathways need to be explored. Third, the existing observational evidence from MICs may be biased from residual confounding by factors which may affect both school-entry age and health such as unmeasured psychological or socio-cultural factors. Similarly, there may be confounding by reverse causality, particularly since the health of children likely also affects their school-entry age (Glewwe & Jacoby, 1995). Stunting and anemia, for example, are widespread health concerns among children in low-resource settings and may affect school entry and progression (Bobonis et al., 2006; Gansaonré et al., 2022). This is also applicable in terms of neurodevelopmental conditions among children, beyond ADHD symptoms, that invariably lead to stigma and delayed school-entry age (Bakare & Munir, 2011). More (quasi-)experimental studies are needed to establish causal inference in LMICs. Fourth, all studies relied on quantitative designs. This small but growing field of research may benefit from additional qualitative and mixed-methods designs to better understand the meaning and context of statistical associations and support the design of interventions that can help offset potential disadvantages stemming from school-entry cut-off dates (e.g., through tailored tutoring support) (Brannen, 2005). Fifth, existing studies from MICs used outcomes reported by parents or teachers (Table 2), which are vulnerable to reporting bias. Future studies could, for instance, use molecular and physiological biomarker data to determine the role of relative age in school and health outcomes (De Neve et al., 2023). This is likely to be particularly salient in distinguishing social from neurobiological effects by group-based differences (e.g., by gender as evidenced in HIC studies) in terms of school-entry age and health and behavior outcomes. Sixth, parents may manipulate the timing of births based on school-entry age policies (Deming & Dynarski, 2008). In Japan and China, for example, parents systematically time births relative to the school entry cut-off (Huang et al., 2020; Shigeoka, 2015). This may be more of a concern in settings where birthdays are less precisely recorded, could be more easily manipulated, or where institutional capacity to implement and enforce strict school-entry age policies may be relatively more limited. Little is known about whether these parental responses to school-entry age policies are more heterogenous in the context of LMICs compared to HICs. Similarly, seasonal variation in nutrient availability (e.g., due to harvest times or seasonal infectious diseases) may also affect fetal development variably in LMICs, with potential impacts across the life course (Almond & Currie, 2011). These early exposures may offer alternate explanations for health differences attributed to school-entry age. Seventh, the concepts of school-entry age, relative age for grade (Peña, 2017, 2020; Peña & Duckworth, 2018), “social age” (Skirbekk et al., 2004), and grade progression in school reflect complex cultural realities and may be challenging to capture empirically. Greater clarity in defining the concept and broader context of school-entry age may further reduce conceptual ambiguity (Barakat & Bengtsson, 2017).

Box 2 **Opportunities for future research to assess school-entry age and health in LMICs**

● Despite much evidence on school-entry age and human capital development from high-income settings, little evidence is available from lower-resource settings, particularly from low-income and lower middle-income countries.
● No studies assessed infectious and most non-communicable diseases as health outcomes, which are pressing health concerns in LMICs, and only one study assessed the effects of school-entry age on malnutrition.
● Existing studies mostly used self-reported outcomes as opposed to, for example, molecular or physiological biomarkers and no studies assessed health outcomes beyond ages 22 years (e.g., health outcomes and behaviors in adulthood).
● Additional qualitative, mixed-methods and (quasi-)experimental research may inform strategies to offset potential disadvantages stemming from school-entry cut-off dates (e.g., tailored tutoring support)
● School-entry age and relative age for grade are challenging to capture empirically and greater clarity in defining the concept and context of school-entry age in low-resource settings may reduce conceptual ambiguity.

Alt-text: Box 2

## 5 Conclusion

School-entry age may have important health implications across the life course, but relatively little is known about its effects in LMICs. Based on a scoping review of the published literature across a wide range of fields, we document adverse neurodevelopmental outcomes (Caye et al., 2020), earlier sexual debut and family formation (Caudillo, 2019; Nguyen & Lewis, 2019), and changes in non-communicable disease risk factors (Caudillo, 2019) as effects of starting school early relative to peers in MICs. Although most studies identified negative health consequences from starting school earlier, the evidence for nutritional outcomes was mixed (Levasseur, 2022). No studies were identified from low-income countries. Further research is needed to investigate the health impacts of starting school early, whether and how these effects persist in adulthood, and to inform strategies that can offset potential disadvantages stemming from school-entry cut-off dates. Moreover, in addition to the health effects of school-entry age, this research relates to several other issues. For example, the question arises as to what motivates parents to send their children to school on time (or not), how these motivations are formed, and which factors may influence these parental decisions. Parents may enroll children as soon as possible to reduce the financial and opportunity costs associated with childcare. Conversely, however, parents may also seek to postpone school enrolment as a response to potential developmental delays resulting from adverse exposures during early childhood (Glewwe & Jacoby, 1995). This review may serve as a foundation for future empirical research on school-entry age and health in the context of poverty.

## Contributors

JL and JWDN conceived and designed the study. JL conducted the analyses under the guidance of all co-authors. JL and JWDN wrote the first draft of the report. All co-authors reviewed and contributed important revisions to the report. All authors approved the final submitted version of the report. The corresponding author attests that all listed authors meet authorship criteria and that no others meeting the criteria have been omitted. JL and JWDN had full access to all the data in the study and had final responsibility for the decision to submit for publication.

## Funding

JWDN was supported by the 10.13039/501100000780European Commission (825823); German Research Foundation (405898232); and NICHD of 10.13039/100000002NIH (R03-HD098982). For the publication fee, we acknowledge financial support by the 10.13039/501100001659German Research Foundation within the funding programme “Open Access Publikationskosten” as well as by Heidelberg University. The funders had no role in study design, data collection and analysis, decision to publish, or preparation of the manuscript.

## Ethical statement

This scoping review of the existing published literature does not require ethics approval since no human participants were involved.

## Declaration of competing interest

We declare that we have no competing interests.

## Data Availability

No data was used for the research described in the article.
