# Supplementary material for: The effect of school-entry age on health is understudied in low- and middle-income countries: A scoping review and future directions for research
Source: SSM Popul Health. 2023 May 2;22:101423. doi: 10.1016/j.ssmph.2023.101423 (PMC10200977; doi:10.1016/j.ssmph.2023.101423)
Supplement: Multimedia component 1 [file mmc1.docx]

**Appendix A Supplementary data**

This supplementary webappendix provides additional data for the manuscript, entitled “*The effect of school-entry age on health is understudied in low- and middle-income countries: A scoping review and future directions for research*”, including:

[Table S1. Search history for Figure 1 2](#_Toc134433557)

[Table S2. Search history in PubMed 3](#_Toc134433558)

[Table S3. Search history in ERIC 4](#_Toc134433559)

[Table S4. Search history in EconLit 5](#_Toc134433560)

[Table S5. Search history in EBSCO 6](#_Toc134433561)

[Table S6. Search history in Google Scholar 7](#_Toc134433562)

[Table S7. Journal impact and field of included studies 8](#_Toc134433563)

## Table S1. Search history for Figure 1

| **PubMed: Search Terms** | | **Results** |
| --- | --- | --- |
| <https://pubmed.ncbi.nlm.nih.gov/> (Search date: 22.09.2022) | | |
| ***Low- and middle-income countries***  (Total population:  6.57 billion in 2021) | (“school entry age” OR “school starting age” OR "age at school entry" OR "age-at-school-entry" OR "birthdate effect" OR "age factors"[mesh] OR "relative age for grade" OR "month of school enrollment" OR "young-for-grade" OR "old-for-grade" OR "month of birth" OR “birthmonth”) AND ("education"[Mesh] OR "Schools"[Mesh] OR "primary education" OR "primary school") AND ("Low income countries" OR "Middle income countries" OR "Low and middle income countries" OR "low resource settings" OR "Developing Countries" OR “Africa” OR “Asia” OR “South America” OR “Central America” OR “Eastern Europe” OR “Middle East” OR “Caribbean” OR “China” OR “India” OR “Indonesia” OR “Pakistan” OR “Brazil” OR “Nigeria” OR “Bangladesh” OR “Mexico” OR “Russia” OR “Ethiopia” OR “Philippines” OR “Egypt” OR “Vietnam” OR “DR Congo” OR “Turkey" OR “Iran” OR “Thailand” OR “Tanzania” OR “South Africa”) AND "Humans"[Mesh] NOT letter [pt] NOT Case Reports [pt] NOT Editorial [pt] NOT Historical Article [pt] NOT Meta-Analysis [pt] NOT Review [pt] NOT Comment [pt] | 3,603 |
| ***High-income countries***  (Total population:  1.24 billion in 2021) | (“school entry age” OR “school starting age" OR "age at school entry" OR "age-at-school-entry" OR "birthdate effect" OR "age factors"[mesh] OR "relative age for grade" OR "month of school enrollment" OR "young-for-grade" OR "old-for-grade" OR "month of birth" OR “birthmonth”) AND ("education"[Mesh] OR "Schools"[Mesh] OR "primary education" OR "primary school") AND ("High income countries" OR "Developed Countries" OR “North America” OR “Europe” OR “America” OR “United Kingdom” OR “Canada” OR “United States” OR “Germany” OR “France” OR “Denmark” OR “Norway” OR “Austria” OR “Ireland” OR “Japan” OR “Belgium” OR “Spain” OR “Sweden” OR “Switzerland” OR “New Zealand” OR “Australia” OR “Finland” OR “Norway”) AND "Humans"[Mesh] NOT letter [pt] NOT Case Reports [pt] NOT Editorial [pt] NOT Historical Article [pt] NOT Meta-Analysis [pt] NOT Review [pt] NOT Comment [pt] | 11,153 |

*Notes:* Total population estimates were obtained from the World Bank Development Indicators database.

## Table S2. Search history in PubMed

| **PubMed: Search Terms (all years)** | | **Results** |
| --- | --- | --- |
| <https://pubmed.ncbi.nlm.nih.gov/> (Search date: 10.08.2022) | | |
| Concept 1 (school entry age) | ("school entry age" OR "school starting age" OR “age at school entry" OR "age-at-school-entry" OR "birthdate effect" OR "age factors"[mesh] OR "relative age for grade" OR "month of school enrollment" OR "young-for-grade" OR "old-for-grade" OR "month of birth" OR “birthmonth”) | 563,605 |
| Concept 2 (primary education) | ("education"[Mesh] OR "Schools"[Mesh] OR "primary education" OR "primary school") | 892,051 |
| Concept 3 (LMICs) | ("Low income countries" OR "Middle income countries" OR "Low and middle income countries" OR "low resource settings" OR "Developing Countries" OR “Africa” OR “Asia” OR “South America” OR “Central America” OR “Eastern Europe” OR “Middle East” OR “Caribbean” OR “China” OR “India” OR “Indonesia” OR “Pakistan” OR “Brazil” OR “Nigeria” OR “Bangladesh” OR “Mexico” OR “Russia” OR “Ethiopia” OR “Philippines” OR “Egypt” OR “Vietnam” OR “DR Congo” OR “Turkey" OR “Iran” OR “Thailand” OR “Tanzania” OR “South Africa”) | 5,302,055 |
| Concept 4 (type of study) | "Humans"[Mesh] NOT letter [pt] NOT Case Reports [pt] NOT Editorial [pt] NOT Historical Article [pt] NOT Meta-Analysis [pt] NOT Review [pt] NOT Comment [pt] | 14,638,365 |
| #1 AND #2 AND #3 AND #4 | ("school entry age" OR "school starting age" OR "age at school entry" OR "age-at-school-entry" OR "birthdate effect" OR "age factors"[mesh] OR "relative age for grade" OR "month of school enrollment" OR "young-for-grade" OR "old-for-grade" OR "month of birth" OR “birthmonth”) AND ("education"[Mesh] OR "Schools"[Mesh] OR "primary education" OR "primary school") AND ("Low income countries" OR "Middle income countries" OR "Low and middle income countries" OR "low resource settings" OR "Developing Countries" OR “Africa” OR “Asia” OR “South America” OR “Central America” OR “Eastern Europe” OR “Middle East” OR “Caribbean” OR “China” OR “India” OR “Indonesia” OR “Pakistan” OR “Brazil” OR “Nigeria” OR “Bangladesh” OR “Mexico” OR “Russia” OR “Ethiopia” OR “Philippines” OR “Egypt” OR “Vietnam” OR “DR Congo” OR “Turkey" OR “Iran” OR “Thailand” OR “Tanzania” OR “South Africa”) AND "Humans"[Mesh] NOT letter [pt] NOT Case Reports [pt] NOT Editorial [pt] NOT Historical Article [pt] NOT Meta-Analysis [pt] NOT Review [pt] NOT Comment [pt] | 3,603 |

## Table S3. Search history in ERIC

| **ERIC: Search Terms (all years)** | | **Results** |
| --- | --- | --- |
| <https://eric.ed.gov/> (Search date: 10.08.2022) | | |
| Descriptor | School entry age | 948 |
| Filter | Peer-reviewed only | 328 |

## Table S4. Search history in EconLit

| **EconLit: Search Terms (all years)** | | **Results** |
| --- | --- | --- |
| <https://www.aeaweb.org/econlit/> (Search date: 10.08.2022) | | |
| Concept 1 (school entry age) | (“school entry age” OR “age at school entry” OR “age-at-school-entry” OR “birthdate effect” OR “age factors”) | 69 |
| Concept 2 (LMICs) | ("Low income countries" OR "Middle income countries" OR "Low and middle income countries" OR "low resource settings" OR "Developing Countries" OR “Africa” OR “Asia” OR “South America” OR “Central America” OR “Eastern Europe” OR “Middle East” OR “Caribbean” OR “China” OR “India” OR “Indonesia” OR “Pakistan” OR “Brazil” OR “Nigeria” OR “Bangladesh” OR “Mexico” OR “Russia” OR “Ethiopia” OR “Philippines” OR “Egypt” OR “Vietnam” OR “DR Congo” OR “Turkey" OR “Iran” OR “Thailand” OR “Tanzania” OR “South Africa”) | 419,919 |
| Concept 3 (health outcomes) | (“developmental outcomes” OR “health” OR “infectious diseases” OR “non-communicable diseases” OR “violence” OR “injuries” OR “biomarkers” OR “fertility” OR “mortality” OR “morbidity” OR “socioemotional development” OR “teen pregnancy” OR “mental health” OR “risk behaviors” OR “maternal and child health” OR “anthropometric measurements” OR “healthcare utilization”) | 155,209 |
| #1 AND #2 AND #3 | (“school entry age” OR “age at school entry” OR “age-at-school-entry” OR “birthdate effect” OR “age factors”) AND ("Low income countries" OR "Middle income countries" OR "Low and middle income countries" OR "low resource settings" OR "Developing Countries" OR “Africa” OR “Asia” OR “South America” OR “Central America” OR “Eastern Europe” OR “Middle East” OR “Caribbean” OR “China” OR “India” OR “Indonesia” OR “Pakistan” OR “Brazil” OR “Nigeria” OR “Bangladesh” OR “Mexico” OR “Russia” OR “Ethiopia” OR “Philippines” OR “Egypt” OR “Vietnam” OR “DR Congo” OR “Turkey" OR “Iran” OR “Thailand” OR “Tanzania” OR “South Africa”) AND (“Developmental outcomes” OR “health returns” OR “biomarkers” OR “fertility” OR “mortality” OR “morbidity” OR “socioemotional development” OR “teen pregnancy” OR “mental health” OR “risk behaviors”) | 11 |

## Table S5. Search history in EBSCO

| **ESBCO: Search Terms (all years)** | | **Results** |
| --- | --- | --- |
| <https://web-s-ebscohost-com.ezp-prod1.hul.harvard.edu/ehost/search/advanced?vid=0&sid=f7ae3604-bbbf-4586-af1d-df9316a67d10%40redis> (Search date: 08.08.2022) | | |
| Concept 1 (school entry age) | school entry age or relative age for grade | 262 |
| Concept 2 (LMICs) | Low-income countries or developing countries or middle-income countries or Africa or Asia or South America or Central America or Eastern Europe or Middle East | 925,104 |
| Concept 3 (health outcomes) | health or development or illness or disease or mortality or non-communicable diseases or infectious diseases | 10,131,780 |
| #1 AND #2 AND #3 | School entry age or relative age for grade AND Low-income countries or developing countries or middle-income countries or Africa or Asia or South America or Central America or Eastern Europe or Middle East AND health or development or illness or disease or mortality or non-communicable diseases or infectious diseases | 34 |

## Table S6. Search history in Google Scholar

| **Google Scholar: Search Terms (all years)** | | **Results** |
| --- | --- | --- |
| <https://scholar.google.com/> (Search date: 08.08.2022) | | |
| Concept 1 (school entry age) | school starting age or relative age for grade | 2,050,000 |
| Concept 2 (LMICs) | low-income countries or developing countries or middle-income countries | 823,000 |
| Concept 3 (health outcomes) | health or development or illness or disease | 4,050,000 |
| #1 AND #2 AND #3 | (“school starting age” OR "relative age for grade") AND ("low-income countries" OR "developing countries" OR "middle-income countries") AND (health OR development OR illness OR disease) | 943 |

| **Google Scholar: Search Terms (all years)** | | **Results** |
| --- | --- | --- |
| <https://scholar.google.com/> (Search date: 28.03.2023) | | |
| Concept 1 (school entry age) | school entry age or relative age for grade | 1,840,000 |
| Concept 2 (LMICs) | low-income countries or developing countries or middle-income countries | 953,000 |
| Concept 3 (health outcomes) | health or development or illness or disease | 5,470,000 |
| #1 AND #2 AND #3 | ("school entry age" OR "relative age for grade") AND ("low-income countries" OR "developing countries" OR "middle-income countries") AND (health OR development OR illness OR disease) | 1,210 |

*Notes:* We used an alternative search strategy using the keyword “school-entry age” instead of “school starting age”, with similar results. Using this alternative search strategy, no additional eligible studies were identified.

## Table S7. Journal impact and field of studies included in scoping review

| **Authors** | **Journal** | **Source-Normalized Impact per Paper** | **CiteScore percentile rank in field** |
| --- | --- | --- | --- |
| Caye et al. (2020) | *Journal of the American Academy of Child & Adolescent Psychiatry* | 3.594 | 99^th^ in Developmental and Educational Psychology |
| Caudillo (2019) | *Demography* | 2.432 | 95^th^ in Demography |
| Gökçe et al. (2017) | *Journal of Developmental and Behavioral Pediatrics* | 1.251 | 66^th^ in Pediatrics, Perinatology and Child Health |
| Gümüş et al. (2021) | *Journal of Experimental and Clinical Medicine (Turkey)* | 0.026 | 0^th^ in General Biochemistry, Genetics and Molecular Biology |
| Levasseur (2022) | *Economics & Human Biology* | 1.301 | 80^th^ in Economics, Econometrics and Finance (miscellaneous) |
| Nguyen et al. (2019) | *Population Research and Policy Review* | 1.091 | 80^th^ in Demography |
| Öner et al. (2018) | *Eurasia Journal of Mathematics, Science and Technology Education* | 1.424 | 87^th^ in Education |
| Öner et al. (2019) | *Scandinavian Journal of Psychology* | 1.118 | 77^th^ in Arts and Humanities (miscellaneous) |

*Notes:* Data extracted from Scopus Preview (2023) (<https://www.scopus.com/sources.uri>)
